# Surface charge-dependent effects of BSA-curcumin nanoparticles combined with amikacin in antimicrobial photodynamic therapy: A pilot In Vivo study of multidrug-resistant Pseudomonas aeruginosa skin infection

**DOI:** 10.1007/s10103-026-05054-5

**Published:** 2026-10-06

**Authors:** Isabelle Ribeiro Brambilla, Eloíse Gwyneth Lopes Oliveira, Mariany Fernandes da Silva Gravito de Carvalho, Isabelle Ferreira, Tassia Joi Martins, Jessica Aparecida Ribeiro Ambrósio, Andreza Ribeiro Simioni, Juliana Guerra Pinto, Juliana Ferreira-Strixino

**Affiliations:** 1 Laboratory of Photobiology Applied to Health - PhotoBioS, Universidade do Vale do Paraíba, São José dos Campos, Brazil; 2 Micro and Nanoparticle Systems Laboratory, Universidade do Vale do Paraíba, São José dos Campos, Brazil

**Keywords:** *Pseudomonas aeruginosa*, Photodynamic therapy, Curcumin, Albumin nanoparticles, Amikacin, Skin infection

## Abstract

Skin infections caused by multidrug-resistant (MDR) *Pseudomonas aeruginosa* represent a significant therapeutic challenge because of high bacterial virulence and increasing antibiotic resistance. In this context, alternative strategies—such as nanotechnology and photodynamic therapy (PDT)—that can be combined with antibiotic therapy are being investigated as promising approaches for treating these infections. This study evaluated the therapeutic response of PDT mediated by anionic and cationic bovine serum albumin nanoparticles (NanoBSA) containing curcumin, in association with amikacin, in a murine model of cutaneous infection by MDR P. aeruginosa. The study was structured as a pilot trial involving 22 BALB/c mice infected and treated with free or nanoencapsulated curcumin and amikacin, followed by irradiation at 450 ± 10 nm. Blood and skin samples from the infected area were collected to analyze leukocyte profiles, serum inflammatory cytokines, and histopathological changes, and to measure changes in lesion area. The results demonstrated that the combination of PDT, NanoBSA, and amikacin promoted a greater reduction in lesion area, particularly with the anionic formulation. Leukocyte analysis indicated modulation of the inflammatory response, showing a more balanced profile in groups treated with amikacin, especially with the anionic formulation. Cytokine evaluation also showed modulation of the inflammatory response, most evident with the cationic formulation combined with amikacin. Histological findings showed improved tissue reorganization and structural preservation in groups undergoing combination therapy, with the anionic NanoBSA formulation standing out. Taken together, the results suggest that the combination of TFD, nanoencapsulated curcumin, and amikacin has therapeutic potential for treating cutaneous infections caused by MDR P. aeruginosa by promoting improved tissue repair and modulating the inflammatory response. Bacterial load was not quantified in the present study, which constitutes a limitation. Future studies should address this assay to confirm antimicrobial efficacy and help elucidate the underlying mechanisms.

**Supplementary Information:**

The online version contains supplementary material available at https://doi.org/10.1007/s10103-026-05054-5.

## Introduction

Infections caused by multidrug-resistant bacteria represent a major global health threat because of high mortality rates and the limited effectiveness of current treatments, largely due to resistance to conventional antibiotics such as cephalosporins, fluoroquinolones, and carbapenems. Among the microorganisms of greatest clinical significance, the ESKAPE group comprises *Enterococcus faecium*, *Staphylococcus aureus*, *Klebsiella pneumoniae*, *Acinetobacter baumannii*, *Pseudomonas aeruginosa*, and *Enterobacter* spp., which are frequently associated with nosocomial infections and exhibit multiple mechanisms of antimicrobial resistance [11, 12, 19], Miller and Arias [27], Aguilar-Salazar et al. [3]. Among these pathogens, *P. aeruginosa* is particularly challenging because of the coexistence of resistance mechanisms such as the production of β-lactamases, carbapenemases, and AmpC, reduced outer membrane permeability, loss or modification of porins, particularly OprD, overexpression of efflux systems, and biofilm formation, which contribute to reduced susceptibility to several antimicrobial classes [3].

The increasing prevalence of multidrug-resistant strains makes treating bacterial infections increasingly challenging, while adverse effects and toxicity may further limit the clinical use of antimicrobial agents [8, 33]. Amikacin, an aminoglycoside with significant activity against Gram-negative bacilli, including *P. aeruginosa*, also presents limitations related to poor gastrointestinal absorption and the risk of nephrotoxicity and ototoxicity. Consequently, researchers have investigated local administration and combination therapies to enhance antimicrobial activity at the infection site. Studies have demonstrated amikacin activity against multidrug-resistant isolates of *P. aeruginosa* and promising results from its combination with other antimicrobial approaches, including photodynamic therapy [14, 25, 36].

In this context, antimicrobial photodynamic therapy (aPDT) has emerged as a potential therapeutic alternative or adjuvant to conventional antibiotics. aPDT relies on the interaction of a photosensitizer (PS) with light at an appropriate wavelength in the presence of molecular oxygen, generating reactive oxygen species that induce oxidative damage to target cells. Its advantages include activity against multidrug-resistant strains and the possibility of repeated applications with a lower likelihood of selecting for resistance due to its multifaceted mechanism of action [13, 15, 45]. However, its therapeutic efficacy may be affected by oxygen availability, light penetration, and the physicochemical properties of the PS, including low aqueous solubility, instability, and poor bioavailability [17, 22, 42, 43].

Among the PSs investigated for aPDT, curcumin, a natural polyphenolic compound, stands out because of its photobiological activity and photodynamic potential. Nevertheless, its application is limited by its short biological half-life, unfavorable pharmacokinetic profile, low bioavailability, poor aqueous solubility, and physicochemical instability Kah et al. [18], Pourhajibagher et al. [35]. Nanotechnology-based delivery systems have therefore been investigated to overcome these limitations, as nanocarriers can incorporate photosensitizers, improve their physicochemical properties, and facilitate their application in aPDT [17, 22, 42, 43].

Among these systems, bovine serum albumin (BSA)-based nanoparticles are promising carriers for curcumin because BSA is biocompatible and can improve the solubility and biological activity of PSs. Importantly, the physicochemical properties of BSA nanoparticles can vary according to their surface charge, which may directly influence their interactions with biological and microbial systems and, consequently, their performance in antimicrobial photodynamic applications [9, 29, 34, 42, 43].

Although curcumin-loaded BSA nanoparticles have demonstrated antimicrobial photodynamic potential [34], *in vivo* studies evaluating how surface charge modulation affects local and systemic therapeutic responses remain scarce, particularly in multidrug-resistant bacterial infections.

Therefore, investigating curcumin-loaded BSA nanoparticles with different surface charges may provide a better understanding of how nanostructural properties influence the therapeutic response to aPDT and whether their combination with conventional antibiotics can enhance antimicrobial treatment.

Based on this rationale, this study aimed to evaluate the effects of aPDT mediated by curcumin encapsulated in BSA nanoparticles with different surface charges, alone or in combination with amikacin, in an *in vivo* model of cutaneous infection caused by a multidrug-resistant strain of *P. aeruginosa*.

## Methodology

### Preparation of formulations and application

Curcumin encapsulated in cationic (NanoBSA +) and anionic (NanoBSA-) BSA nanoparticles was prepared using the desolvation method, as described by Marcolino et al. [24]. The nanoparticles were characterized via UV–Vis spectroscopy (300–500 nm),dynamic light scattering (DLS) to determine particle size, polydispersity index (PDI), and zeta potential; and scanning electron microscopy (SEM) for morphological analysis.

Two base cream formulations were prepared: one containing 1.5% free curcumin (PDT Pharma, Cravinhos, SP, Brazil)—supplied by the São Carlos Institute of Physics (USP)—and another containing NanoBSA + and NanoBSA − , both formulated at 1.5% for topical application.

For the combination treatment, amikacin was added separately to the topical formulation at 30 µg/mL. This concentration was selected based on previous in vitro experiments with bacterial biofilms, using the resazurin metabolic activity assay. Among the concentrations evaluated, 60 µg/mL resulted in the lowest metabolic activity; 30 µg/mL was chosen because it is half that value, allowing evaluation of a reduced antibiotic concentration in combination with PDT. This strategy was adapted from Akhtar et al. [4].

With regard to cytocompatibility, the same formulations of cationic and anionic BSA-curcumin nanoparticles used in the present study were previously evaluated in vitro in RAW 264.7 macrophages using the trypan blue exclusion assay in the study by Marcolino et al. [24].

### Quantification of curcumin in topical formulations

The curcumin concentration in the topical formulations was quantified by UV–Vis spectrophotometry using a method adapted from Saka et al. [39]. To construct the calibration curve, curcumin solutions in ethanol were prepared with concentrations ranging from 1 to 5 µmol/L. Absorption spectra were recorded on a Varian Cary 50 spectrophotometer using a quartz cuvette with a 1.0 cm optical path length. The molar absorptivity coefficient was determined from the absorbance and concentration values.

The topical formulations analyzed were: free curcumin, NanoBSA − , and NanoBSA + . For sample preparation, the formulations were dispersed in ethanol and ultrasonicated until fully solubilized. The samples were centrifuged, and the supernatants were collected to calculate the curcumin concentration based on the absorbance measured at 430 nm.

### Experimental design

**Fig. 1:**
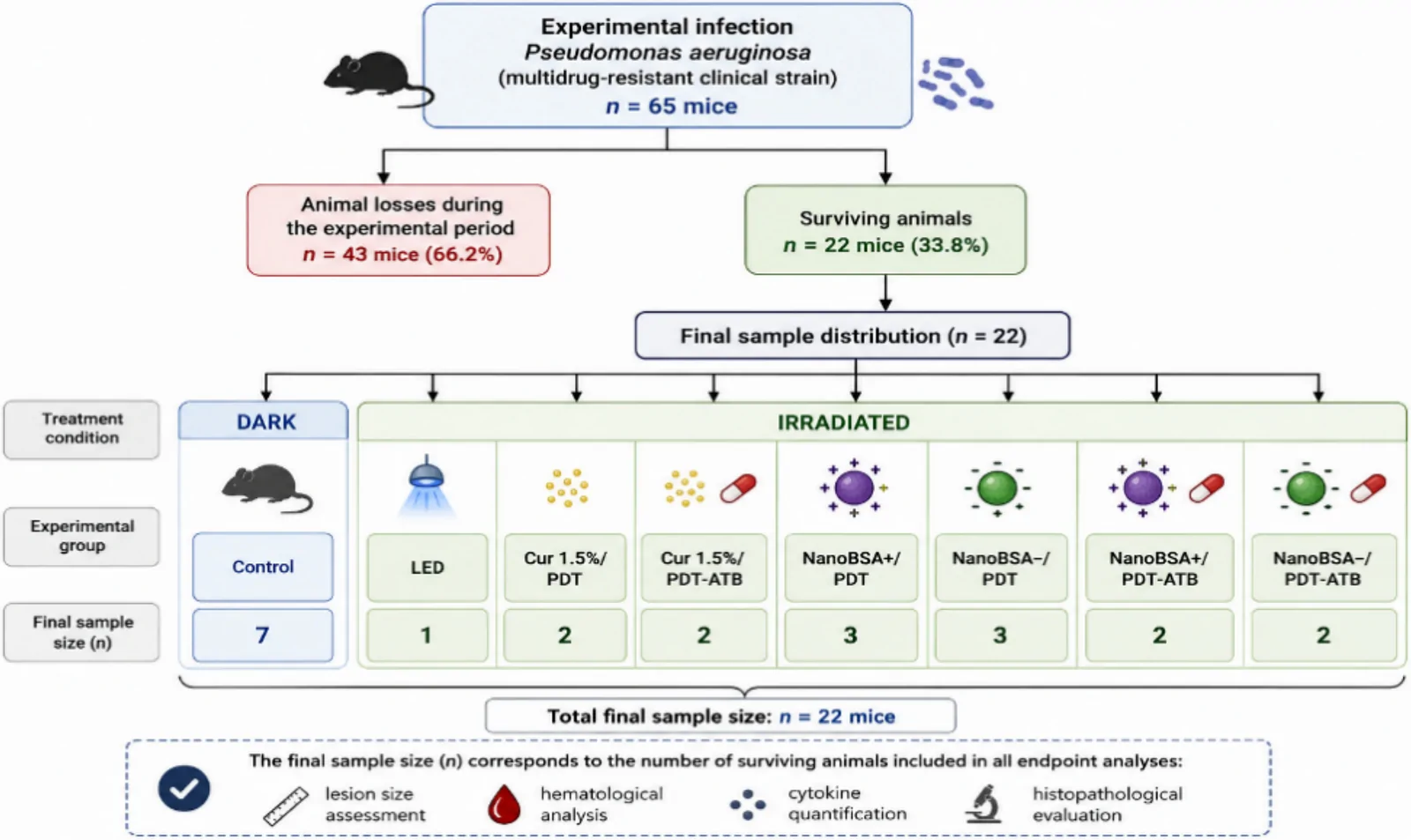
Experimental design, animal losses, and final sample distribution among treatment groups

A total of 65 BALB/c mice were infected with the P. aeruginosa MDR strain; however, after bacterial inoculation, an unexpectedly high mortality rate was observed. After inoculation, the animals were monitored until they had recovered sufficiently from sedation to allow their safe return to the animal facility, where they were kept under appropriate housing conditions. The animals were found dead the morning following inoculation, indicating that death occurred during the 12-h dark cycle. In total, 43 animals died during the experimental period, corresponding to a mortality rate of 66.2%, while 22 animals survived and proceeded to the experimental treatments. Animals that died were not replaced during the study, in adherence to the 3Rs principles. Figure 1 presents the final sample size and the distribution of survivors among the experimental groups. Because of the high mortality rate and the resulting inequality in sample size between groups, the results should be interpreted as exploratory data from an in vivo pilot study (Table 1).

**Table 1:** Experimental groups, treatment conditions, and final sample sizes of BALB/c mice infected with multidrug-resistant *Pseudomonas aeruginosa*

Formulation	Dark	Final sample size (*n*)	Irradiated	Final sample size (*n*)
Without added formulations	Control	7	LED	1
Free curcumin	-	-	Cur 1.5%/PDT	2
Cationic NanoBSA	-	-	NanoBSA + /PDT	3
Anionic NanoBSA	-	-	NanoBSA − /PDT	3
Free curcumin + amikacin	-	-	Cur 1.5%/PDT-ATB	2
Cationic NanoBSA + amikacin	-	-	NanoBSA + /PDT-ATB	2
Anionic NanoBSA + amikacin	-	-	NanoBSA − /PDT-ATB	2

### Acquisition and maintenance of animals

For the experiments, 4-week-old male BALB/c mice were used. The animals were acquired from the Multidisciplinary Center for Biological Research in the Area of ​​Laboratory Animal Science (CEMIB – Unicamp) and kept in the Animal Facility, in appropriate cages, at a controlled temperature of 24 ± 2 °C, simulating a 12-h Circadian cycle with water and food ad libitum and cleaning of the cage, waterer, and food and water replacement carried out 3 times a week.

### Infection of animals

The animals were sedated with 2% xylazine at a dose of 10 mg/kg via peritoneal injection. Subsequently, approximately 0.5 cm of hair was shaved in the dorsal thoracic region, followed by scarification of the area using a sterile scalpel blade. For subcutaneous inoculation, 10 μL of a bacterial solution at 10⁸ CFU/mL, standardized on the McFarland scale, of a multidrug-resistant strain of *P. aeruginosa* was used. After completion of the protocol, the animals were monitored until sedation wore off and then returned to the animal facility.

### Irradiation protocol

After infection, the formulations were applied to the injured area, and after a 10-min incubation period, the region was irradiated using the Elite Olympus (DMC) equipment at a wavelength of 450 nm ± 10 nm, 200 mW/cm2, and a fluence of 50 J/cm2 with continuous pulse emission for 260 s.

The protocol comprised 5 sessions with a 48-h interval between each session, and at the end of each session, the animals were kept away from light in a controlled-lighting environment in the Bioterium. Twenty-four hours after the last session, the animals were euthanized.

### Mouse euthanasia protocol

The animals were euthanized by overdose of combined anesthetics, namely 10% Ketamine and 2% Xylazine. The medication was administered peritoneally, using doses of 300 mg/kg of Ketamine and 30 mg/kg of Xylazine.

### Measuring the injured area

The lesion area in the animals was measured with a digital caliper, in millimeters, before and after infection and at the time of each treatment application. Finally, the lesions were measured to compare the evolution of tissue repair.

### Histopathological analysis

After euthanasia of the animals, skin fragments were collected from the injured area of ​​the sample groups, which were fixed in 10% formalin, dehydrated, cleared, and embedded in paraffin. Sections "Conclusion" µm thick, obtained using a Leica microtome (RM2245), were placed on glass slides and stained with hematoxylin–eosin (H&E). For evaluation, an optical microscope (Leica – DM 2500) was used, and the samples were photographed with a digital image capture system (Leica DF425 camera), with images acquired using Leica Application Suite 4.6.1 software.

### Leukocyte profile

In this study, samples were collected via intracardiac puncture, and the quantitative, qualitative, and morphological characteristics of leukocytes were assessed. The LB Rapid Panoptic staining kit was used, comprising Romanowsky hematological staining. After blood collection, a smear (blood extension) was prepared on a glass slide, dried at room temperature, then submerged in Panoptic 1, 2, and 3, washed in running water, and dried vertically.

The slides were viewed under an optical microscope (Leica DM 2500) and photographed with a digital image capture system (Leica DF425 camera); images were acquired using Leica Application Suite 4.6.1 software. For reading, the leukocyte differential was performed using an absolute count of 100 leukocytes.

### Quantification of cytokines by flow cytometry

Blood samples were collected by intracardiac puncture, placed in microtubes, and centrifuged to obtain serum. Inflammatory cytokines were quantified using the Cytometric Bead Array (CBA) Mouse Inflammation kit (BD Biosciences) on the BD Accuri™ C6 Plus flow cytometer, according to the manufacturer's instructions.

The lyophilized inflammatory standard was reconstituted in 2 mL of Assay Diluent, serially diluted, and the Assay Diluent was used as a negative control. The Capture Bead mix (IL-6, IL-10, IL-12p70, MCP-1, IFN-γ, and TNF) was prepared based on the total number of samples, with 10 μL of each bead added to each.

For the assay, 50 μL of the bead mix and 50 μL of serum were added to each microtube, followed by 50 μL of the detection reagent. The samples were incubated for 2 h at room temperature, protected from light, and then 1 mL of washing buffer was added. The samples were then centrifuged, the supernatant discarded, and the pellet resuspended in 300 μL of washing buffer for flow cytometric analysis.

### Statistical analysis

Due to the high mortality observed following infection, final sample sizes were small and unequal across experimental groups. Therefore, data on lesion area, leukocyte profile, and cytokines were analyzed descriptively and presented as exploratory findings. Histopathological findings were also analyzed qualitatively.

## Results

Higher-than-expected mortality was observed after inoculation with multidrug-resistant *P. aeruginosa*. Although the infection protocol was standardized, losses occurred before the treatment was completed. No replacement animals were included after the study began.

### Curcumin content in topical formulations

The curcumin concentration in the topical formulations was quantified by UV–Vis spectroscopy, with a mean molar absorptivity coefficient of 5.2 × 10^4^ L·mol⁻^1^·cm⁻^1^. Calibration curves and detailed quantification data are presented in the Supplementary Material (Figure S1 and Table S1).

### Evolution of tissue repair in the injured area

**Fig. 2:**
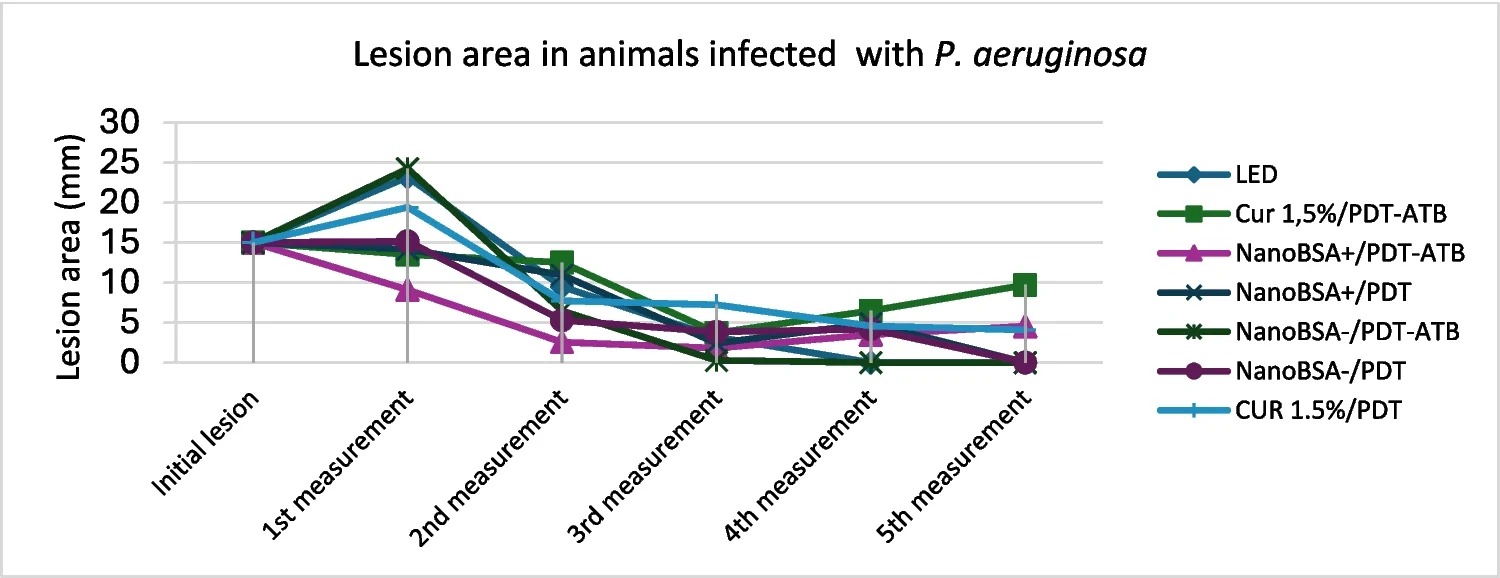
Progression of skin lesion area (mm^2^) over six assessment time points (initial lesion and five subsequent measurements) in animals infected with *P. aeruginosa* after different experimental treatments. Final sample sizes ranged from *n* = 1–3 per group. Data are presented descriptively; no inferential statistical analysis was performed

Figure 2 shows the progression of lesions in the experimental groups. The results show an initial increase in lesioned area in all groups except NanoBSA + /PDT-ATB, followed by a progressive reduction across measurements.

The LED group showed a progressive reduction, demonstrating a partial healing profile, without the same efficiency observed in some treated groups. Among treatments without amikacin, the CUR 1.5%/PDT group showed an oscillating recovery pattern, with a gradual reduction in area and stagnation at the end. The NanoBSA-/PDT and NanoBSA + /PDT groups showed more uniform repair, less variability among samples, and progressive healing.

Groups receiving therapy and antibiotics showed more pronounced healing. The NanoBSA + /PDT-ATB group showed a marked reduction in lesion area in the initial assessments, followed by stabilization in the final measurements, whereas the NanoBSA − /PDT-ATB group showed continuous lesion regression after the initial oscillation, approaching complete repair at the final assessments.

Overall, the descriptive findings suggest a more pronounced reduction in lesion area in some PDT groups, particularly when PDT was combined with amikacin.

### Analysis of the leukocyte profile

**Fig. 3:**
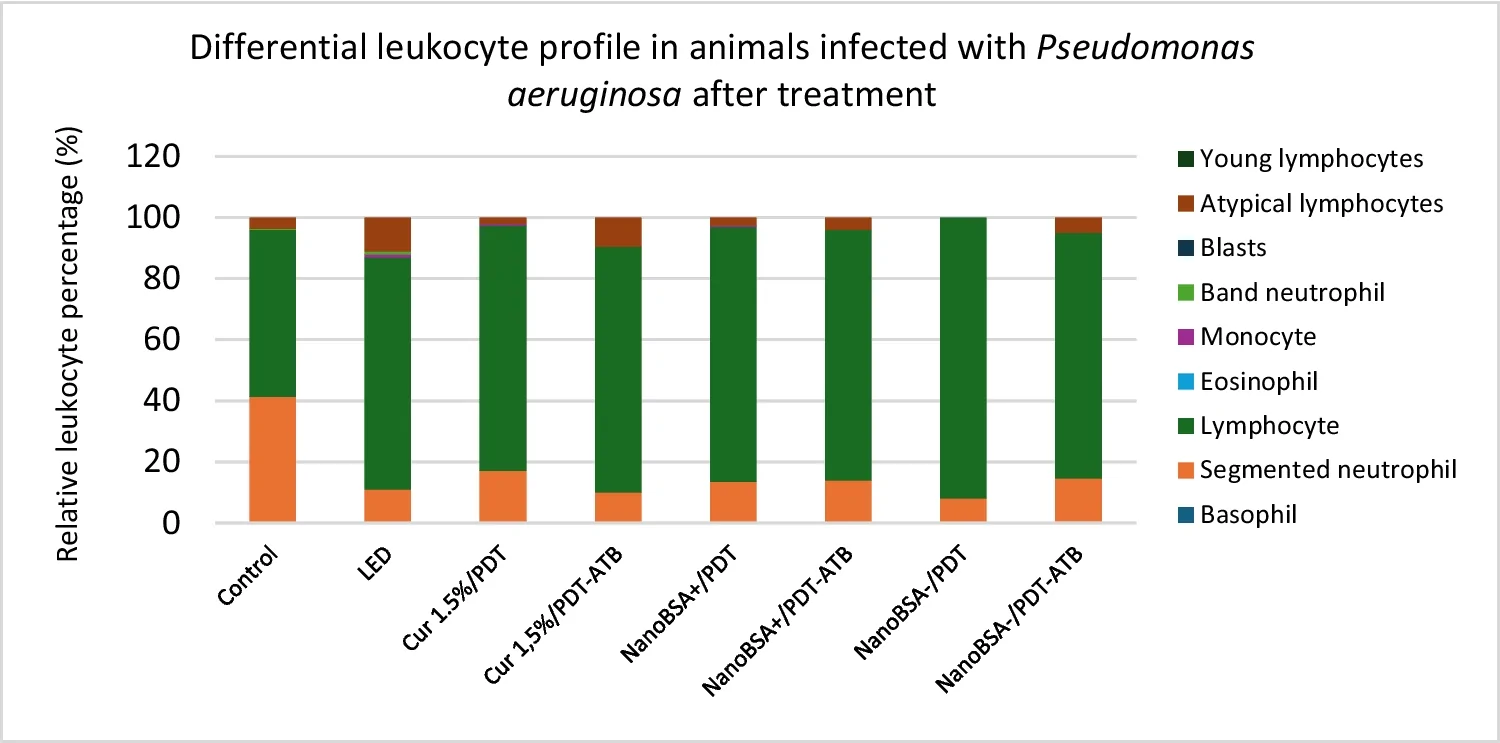
Differential leukocyte profile in BALB/c mice infected with *P. aeruginosa* after treatment. Final sample sizes ranged from *n* = 1–7 per group. Data are presented descriptively; no inferential statistical analysis was performed

The leukogram allowed us to define the hematological profile associated with each treatment. Figure 3 shows the analysis of segmented neutrophils, lymphocytes, eosinophils, monocytes, band neutrophils, atypical lymphocytes, and young lymphocytes in the sample groups.

It was possible to observe in the Control, LED, NanoBSA + /PDT, NanoBSA-/PDT, and Cur 1.5%/PDT groups a predominance of lymphocytes of approximately 70 to 85%, followed by reduced proportions of segmented neutrophils and discrete values ​​of monocytes, eosinophils, and band neutrophils, indicating the presence of a controlled inflammatory process.

In groups with PDT associated with antibiotics (Cur 1.5%/PDT-ATB, NanoBSA + /PDT-ATB; NanoBSA-/PDT-ATB), particularly in the cationic and anionic NanoBSA formulations, the leukocyte profile differed from that of the control samples, with a reduction in the fraction of segmented neutrophils and stabilization of the percentage of mature lymphocytes.

Furthermore, in the groups with cationic and anionic NanoBSA with antibiotics and PDT, a discrete presence of atypical lymphocytes and immature cells was observed, indicating an immunological response without exacerbated activation. These descriptive findings indicate differences in leukocyte profiles among the experimental groups; however, they should be interpreted cautiously given the small and unequal sample sizes.

### Assessment of cytokine production

**Fig. 4:**
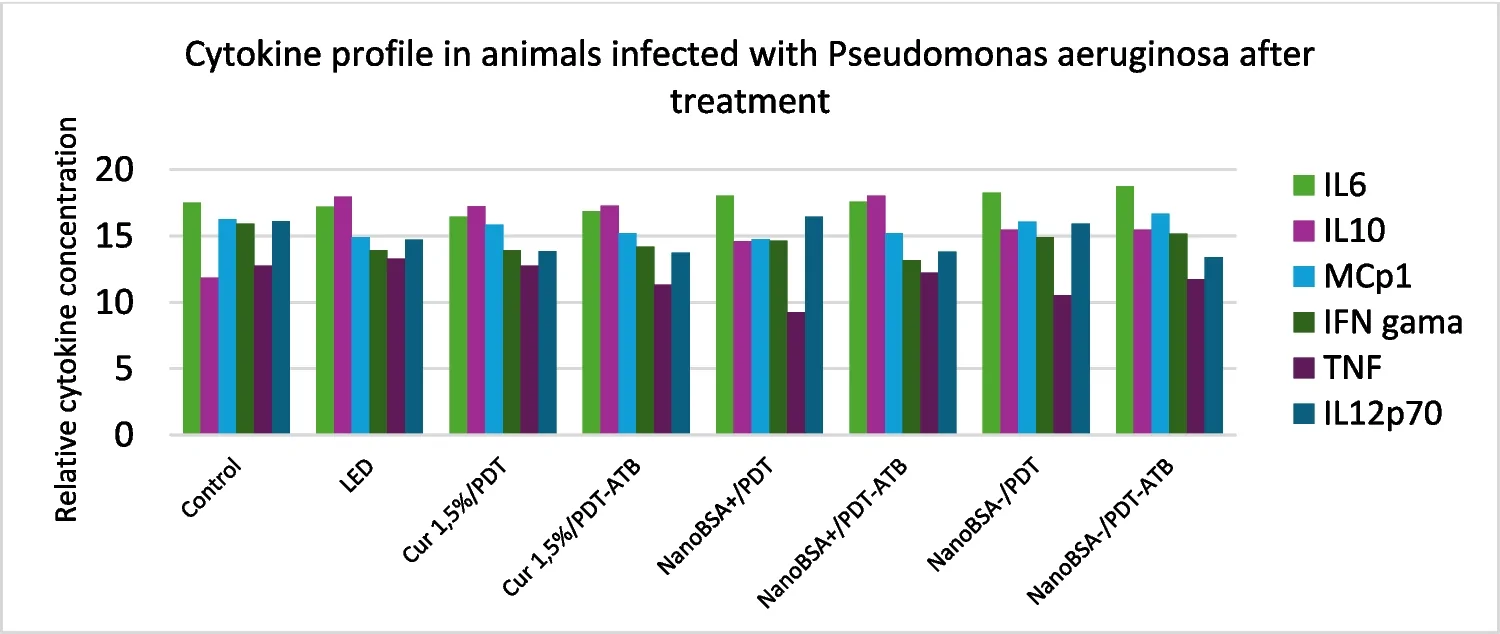
Relative levels of inflammatory cytokines in animals infected with *P. aeruginosa* after treatments with photodynamic therapy mediated by free curcumin or BSA nanoparticles loaded with curcumin, alone or in combination with amikacin. Final sample sizes ranged from *n* = 1–7 per group. Data are presented descriptively; no inferential statistical analysis was performed

Figure 4 shows the cytokine profile analysis, including measurements of IL-6, IL-10, MCP-1, IFN-γ, TNF, and IL-12p70. Based on the results, a homogeneous inflammatory pattern was observed across the experimental groups; however, slight variations were noted after treatment.

Stable levels of the cytokines IL-6, MCP-1, TNF, and IL-12p70 were observed in all experimental groups, along with an increase in IL-10. This finding indicates a moderate systemic inflammatory response with simultaneous activation of pro-inflammatory and regulatory pathways.

In the LED group, the action of light alone on cytokine production was verified, showing a balanced profile with a similar distribution between inflammatory and regulatory mediators. The groups treated with PDT mediated by the combination of NanoBSA and amikacin formulations showed slight modulation of the inflammatory response.

The NanoBSA + /PDT-ATB group showed increased IL-6 and MCP-1, with a notable elevation in IL-10 levels, suggesting that the treatment may have reduced systemic inflammation without inducing immune hyperactivation. The anion load showed a slight increase in IFN-γ and TNF. However, these changes were small, and the overall distribution of cytokines was similar across the experimental groups. Thus, *P. aeruginosa* infection showed a more stable inflammatory response and less susceptibility to cytokine modulation in this experimental model.

### Histopathological evaluation

**Fig. 5:**
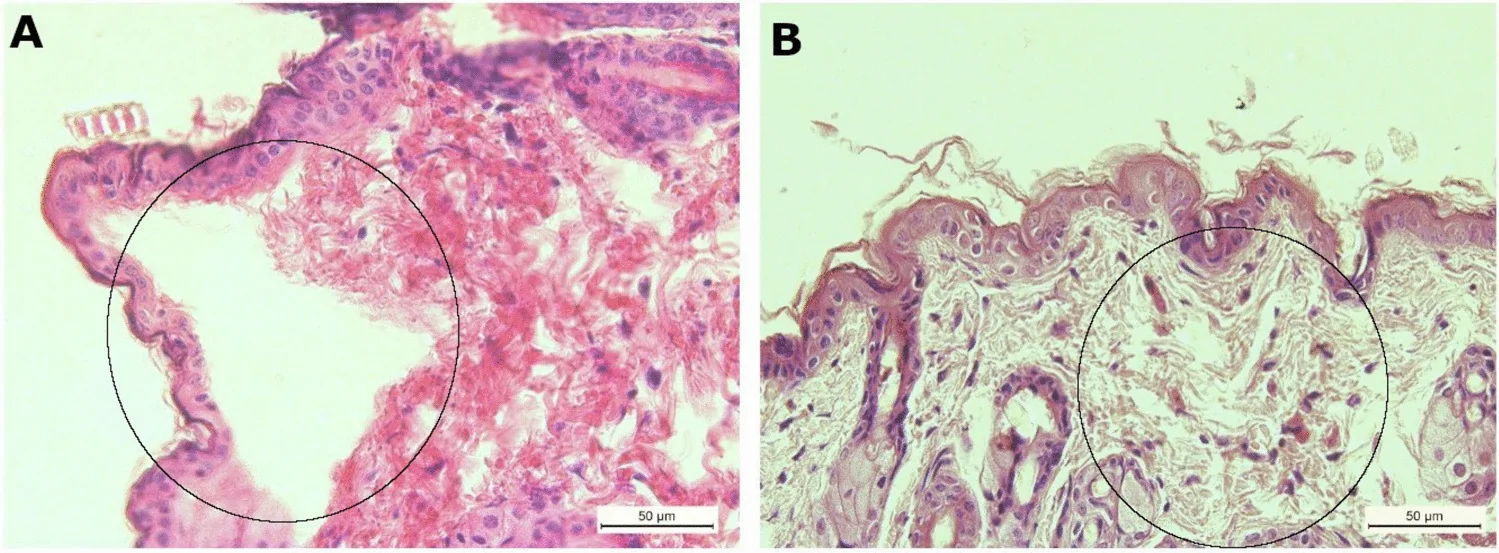
Representative micrographs of skin tissue from animals infected with P. aeruginosa. **A** Control: dermoepidermal separation (circle), without prominent inflammatory infiltrate; **B** LED: mild edema and disorganization of collagen bundles in the superficial dermis, with slight dermoepidermal separation (circle). H&E, 40 ×

In the control group, the epidermis exhibited few cell layers, and the dermis appeared relatively preserved. Perinuclear halos, focal discontinuity between the epidermis and dermis, and loose, poorly aligned collagen fibers were observed in some samples (Fig. 5).

Other samples showed homogeneous nuclei and continuity between the skin layers, indicating heterogeneity in tissue morphology within this group. In the LED group, thickening of the stratum corneum and perinuclear halos in epidermal cells were observed, along with nuclear irregularities and loose collagen fibers in the dermis.

**Fig. 6:**
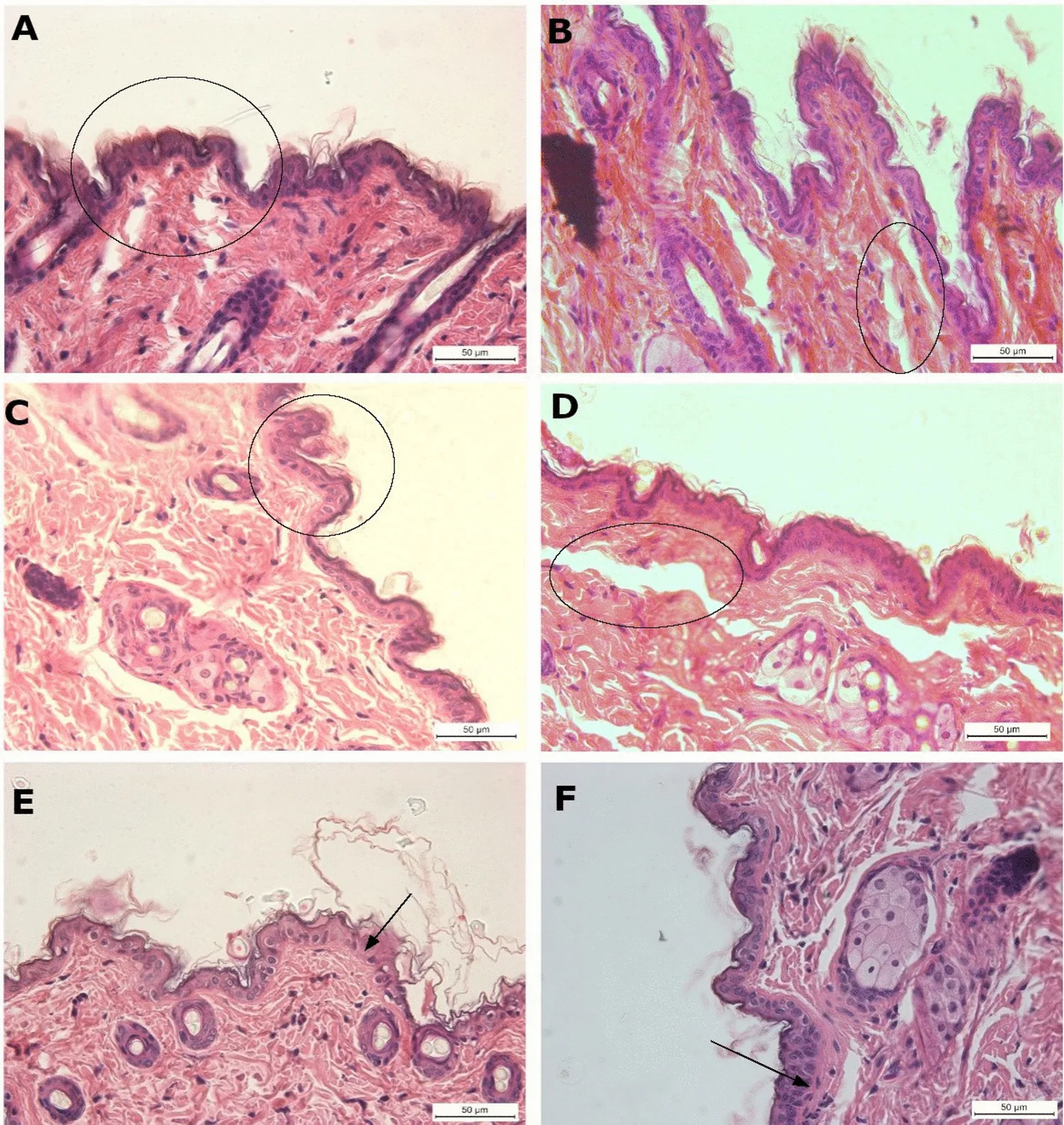
**A** Cur 1.5%: Intracytoplasmic vacuolization of keratinocytes associated with edema (circle); **B** Cur 1.5%/PDT-ATB: Separation of collagen bundles in the superficial dermis, compatible with focal dermal edema (circle); **C** NanoBSA + /PDT: Acanthosis and epidermal projection (circle); **D** NanoBSA + /PDT-ATB: Separation between the epidermis and superficial dermis (circle); **E** NanoBSA − /PDT: Exocytosis of mononuclear inflammatory cells into the epidermis (arrow); and **F** NanoBSA − /PDT-ATB: Mononuclear inflammatory infiltrate in the superficial dermis adjacent to the dermoepidermal junction (arrow). H&E, 40 ×

In the Cur 1.5%/PDT group (Fig. 6), thickening of the stratum corneum, perinuclear halos, nuclear irregularities, and focal discontinuity of dermal collagen fibers were observed. In the amikacin group, hyperkeratosis and discontinuity between the epidermis and dermis were also observed.

The NanoBSA + /PDT group exhibited thickening of the stratum corneum, perinuclear halos, nuclear irregularities, and focal discontinuity between the epidermis and dermis. Loose chromatin and visible nucleoli were observed in some cells. In the amikacin-treated group, a thinner epidermis and localized areas of disorganized dermal collagen fibers were observed. The NanoBSA − /PDT group showed perinuclear halos, nuclear irregularities, and hyperkeratosis, while the dermal architecture remained preserved in the analyzed sections.

In the amikacin group, hyperkeratosis, focal disorganization of dermal collagen fibers, and keratin granules in the epidermis were observed. Overall, the histological sections revealed differences in epidermal organization and dermal morphology among the experimental groups. The histological evaluation was predominantly descriptive and morphological.

## Discussion

The present study provides preliminary *in vivo* evidence that aPDT mediated by curcumin-loaded BSA nanoparticles, particularly when combined with amikacin, may influence the progression of cutaneous lesions caused by multidrug-resistant *P. aeruginosa*. Among the evaluated formulations, NanoBSA − /PDT-ATB showed the most continuous reduction in lesion area, approaching complete repair in the final assessments, whereas NanoBSA + /PDT-ATB showed a marked initial reduction followed by stabilization. In contrast, groups treated without amikacin generally exhibited a less pronounced or more variable repair pattern. Although these findings are descriptive, they suggest that combining aPDT with antibiotic therapy may provide greater therapeutic benefit than either photodynamic treatment alone under the conditions evaluated. Such an effect could result from complementary mechanisms involving oxidative damage induced by aPDT and the antibacterial activity of the antibiotic [5, 32, 37, 40].

The potential benefit of combining photodynamic and antibiotic therapies is supported by previous studies, although the magnitude and direction of this interaction depend on the antimicrobial agent and irradiation parameters. Zangirolami et al. [46], for example, demonstrated different responses when antibiotics were combined with aPDT against *P. aeruginosa*, with gentamicin exhibiting synergistic behavior, whereas ciprofloxacin ranged from synergistic to antagonistic effects. Because gentamicin and amikacin are both aminoglycosides, these findings provide a relevant basis for interpreting the more pronounced lesion regression observed in the groups receiving amikacin in the present study. Nevertheless, because bacterial burden was not quantified, the improvement in lesion area cannot be directly attributed to enhanced bacterial killing and may also reflect modulation of the host inflammatory and tissue-repair responses.

Indeed, the evolution of lesion area should be considered together with the systemic leukocyte profile. Most groups showed lymphocyte predominance, whereas the groups receiving aPDT combined with amikacin, particularly those treated with NanoBSA formulations, exhibited lower proportions of segmented neutrophils and relative stabilization of mature lymphocytes. Since neutrophils represent an important component of the acute response to bacterial infection, the comparatively lower proportion of circulating segmented neutrophils in these groups may suggest a less prominent systemic acute inflammatory component at the time of collection. However, this interpretation should not be understood as evidence of immunosuppression or resolution of infection, because leukocyte trafficking between blood and infected tissue was not directly evaluated. Rather, when considered alongside lesion regression, these findings raise the possibility that the combined treatments were associated with a transition toward a more regulated inflammatory state during tissue repair. The discrete presence of atypical and immature lymphocytes in some treated animals further suggests that immune activity persisted despite the reduction in lesion area.

This interpretation is complemented by the cytokine profile. Serum IL-6, MCP-1, TNF, IFN-γ, and IL-12p70 showed relatively small variations among the experimental groups, whereas IL-10 remained comparatively elevated, indicating coexistence of inflammatory and regulatory signals rather than complete suppression of inflammation. This pattern is biologically relevant when interpreted together with the leukogram. IL-6 and TNF are associated with inflammatory activation, whereas MCP-1 participates in monocyte recruitment to injured tissues; recruited monocytes can subsequently differentiate into macrophages and participate both in antimicrobial responses and in inflammation resolution and tissue repair [2, 47]. Conversely, IL-10 has an important regulatory role and may contribute to limiting excessive inflammatory responses. Therefore, the maintenance of inflammatory mediators together with IL-10 may represent a state in which inflammatory activity required for host defense persists concomitantly with regulatory mechanisms associated with tissue recovery. This interpretation would be consistent with the predominance of lymphocytes and relatively lower proportions of circulating segmented neutrophils observed in several treated groups.

The NanoBSA + /PDT-ATB group deserves particular consideration because it showed a slightly distinct cytokine pattern, with increased IL-6 and MCP-1 accompanied by a notable elevation in IL-10. Rather than indicating simply an increase or decrease in inflammation, this combination may suggest simultaneous activation of inflammatory recruitment and regulatory pathways. MCP-1 could favor recruitment of monocytes/macrophages to the injured tissue, while IL-10 could contribute to controlling excessive inflammatory activation. The coexistence of these signals may help explain why changes in serum cytokines were relatively modest despite progressive lesion repair. Moreover, the cytokine profile represents a systemic measurement at a single endpoint and may not reproduce the concentrations or temporal dynamics of mediators within the local wound microenvironment. Thus, the apparently modest systemic cytokine modulation does not necessarily imply absence of local immunological effects.

Interestingly, the NanoBSA − /PDT-ATB group displayed a somewhat different pattern. This group showed the most continuous lesion regression and a comparatively balanced leukocyte profile, whereas serum cytokine changes were less pronounced, with only slight increases in IFN-γ and TNF. Rather than being contradictory, these findings may suggest that the therapeutic response of the anionic formulation was associated with tissue repair without marked systemic cytokine activation. Xiao et al. [41] reported that anionic nanostructures may be associated with lower nonspecific inflammatory activation and more balanced cellular responses. Although this mechanism was not directly assessed here, it provides one possible explanation for the coexistence of pronounced lesion regression and relatively modest systemic cytokine changes in the NanoBSA − /PDT-ATB group.

Importantly, the histopathological findings provide a local tissue perspective that complements both the leukocyte and cytokine profiles. Histological examination demonstrated heterogeneous epidermal and dermal alterations among the groups, including hyperkeratosis, perinuclear halos, dermoepidermal discontinuity, edema, changes in collagen organization, and mononuclear inflammatory infiltrates. In the representative sections, NanoBSA − /PDT showed exocytosis of mononuclear inflammatory cells into the epidermis, whereas NanoBSA − /PDT-ATB exhibited mononuclear inflammatory infiltrate in the superficial dermis adjacent to the dermoepidermal junction. These findings indicate that reduction in lesion area did not occur in the absence of inflammation. Instead, particularly in the anionic formulation combined with amikacin, tissue repair appears to have progressed while local inflammatory cells remained present.

This coexistence is compatible with the dynamic nature of wound healing, in which inflammation and tissue repair are overlapping rather than mutually exclusive processes [23, 38]. Thus, the presence of mononuclear inflammatory infiltrates in animals showing lesion regression should not necessarily be interpreted as therapeutic failure. A plausible interpretation is that these cells represented ongoing local immune activity during transition toward tissue repair. This hypothesis is supported by the detection of MCP-1, which is involved in monocyte recruitment, and by the systemic predominance of mononuclear cells in the leukocyte profile. Although the present study did not identify the phenotype of cells within the tissue, recruited monocytes and macrophages may participate first in pathogen clearance and inflammatory signaling and subsequently in resolution, extracellular matrix remodeling, and repair. Yang et al. [44] similarly reported changes in inflammatory mediators after photodynamic treatment accompanied by macrophage transition from an M1- to an M2-associated phenotype and progression of wound healing. Therefore, one possible interpretation of the present findings is that aPDT combined with amikacin allowed inflammatory activity to persist while favoring progression toward the reparative phase, particularly in animals treated with NanoBSA − .

The relationship among lesion regression, systemic inflammatory markers, and tissue morphology also suggests that the cationic and anionic formulations may have influenced the host response through different biological pathways [31]. The NanoBSA + formulation combined with amikacin was associated with more evident cytokine modulation, particularly involving IL-6, MCP-1, and IL-10, whereas NanoBSA − /PDT-ATB was associated with more continuous lesion regression and comparatively favorable tissue organization despite the persistence of local mononuclear inflammation. Thus, the findings do not indicate that one surface charge uniformly increased or decreased inflammation. Rather, they suggest that surface charge may influence the balance between systemic inflammatory signaling, local cellular recruitment, and tissue repair. This distinction is relevant because successful treatment of an infected wound depends not only on antimicrobial activity but also on preventing excessive inflammatory damage while preserving the cellular responses required for repair.

The physicochemical characteristics of the nanostructures provide one possible basis for these distinct biological profiles, but they should be interpreted as a mechanistic hypothesis rather than as the central finding of the study. The topical formulations contained relatively similar curcumin concentrations—3.8 µmol/L for NanoBSA − and 2.7 µmol/L for NanoBSA + —and previous studies have shown that BSA nanoparticles containing curcumin maintain suitable photophysical and photochemical properties despite opposite surface charges [34]. Therefore, differences in therapeutic response are unlikely to be explained solely by curcumin concentration and may also involve surface charge-dependent interactions with proteins, cells, and components of the wound microenvironment [1, 30, 41].

Cationic nanoparticles generally show stronger interactions with negatively charged cellular membranes, which may favor cellular association but can also increase nonspecific interactions with biological components and protein adsorption. The resulting *protein corona*—a layer of proteins adsorbed onto the nanoparticle surface—can modify the biological identity of the nanostructure and consequently influence cellular interactions, aggregation, and tissue distribution. In contrast, anionic nanoparticles may exhibit greater colloidal stability and lower nonspecific interactions under some biological conditions. These properties could have contributed to the distinct inflammatory and tissue responses observed between NanoBSA + and NanoBSA − , although nanoparticle biodistribution, cellular uptake, and *protein corona* formation were not directly measured in the present study [6, 7, 20]. Therefore, the physicochemical explanation should be considered supportive of, rather than a substitute for, the biological findings.

The observed relationship between inflammatory modulation and tissue repair is also consistent with studies showing that nanostructured systems can influence the wound microenvironment by modulating inflammatory mediators and promoting processes associated with tissue regeneration. A transition from persistent inflammatory signaling toward a regulated reparative microenvironment may favor extracellular matrix reorganization and dermal remodeling [10, 16]. In addition, nanoencapsulation may help overcome the physicochemical limitations of free curcumin. Curcumin-loaded nanoparticles have previously demonstrated antimicrobial activity against *P. aeruginosa *in vitro and improved repair in murine models of infected wounds [21, 28]. These observations are consistent with the generally more uniform lesion regression observed with NanoBSA formulations compared with free curcumin in the present study.

The combination of nanoencapsulated curcumin with antimicrobial agents may further enhance this therapeutic approach. Medaglia et al. [26] demonstrated that mesoporous silica nanoparticles containing curcumin combined with polymyxin B, under blue LED irradiation at 470 nm, exhibited greater antibacterial and antibiofilm activity against *P. aeruginosa* than free curcumin or antibiotic at equivalent concentrations. Although both the nanocarrier and antibiotic differed from those used in the present study, these findings support the rationale for combining nanoencapsulated photosensitizers with conventional antimicrobials. Taken together with the present lesion, leukocyte, cytokine, and histological findings, this evidence suggests that the benefit of the combined strategy may extend beyond direct antimicrobial effects and involve modulation of the inflammatory and reparative microenvironment. Direct bacterial quantification and local immune characterization will be necessary to test this hypothesis.

The main contribution of the present pilot study is therefore not only the comparison of BSA-curcumin nanoparticles with different surface charges, but the preliminary demonstration that these formulations may be associated with distinct integrated biological responses in vivo. NanoBSA − combined with aPDT and amikacin was associated with the most continuous lesion regression, a comparatively balanced circulating leukocyte profile, and tissue findings compatible with ongoing repair in the presence of local mononuclear inflammatory activity. In contrast, NanoBSA + combined with amikacin showed more evident modulation of systemic cytokines, particularly IL-6, MCP-1, and IL-10. Together, these observations support the hypothesis that surface charge may influence not only nanoparticle behavior but also the relationship between inflammatory regulation and tissue repair during treatment of MDR *P. aeruginosa* infection. This interpretation remains exploratory and should be tested in adequately powered mechanistic studies.

Finally, the findings must be interpreted considering the limitations of this pilot model. The unexpectedly high mortality after MDR *P. aeruginosa* inoculation resulted in small and unequal sample sizes, precluding inferential statistical comparisons and requiring descriptive interpretation of the results. In addition, bacterial burden was not directly quantified, preventing determination of whether lesion regression and inflammatory modulation reflected bacterial clearance, host-response modulation, or both. The absence of local cytokine quantification, immunohistochemical characterization of the inflammatory infiltrate, and assessment of NanoBSA biodistribution and cellular uptake also prevents confirmation of the mechanisms proposed above. Future studies with optimized inoculum, larger and balanced experimental groups, bacterial quantification, longitudinal assessment of inflammatory mediators, and characterization of macrophage and neutrophil populations within the lesion will be important to determine how nanoparticle surface charge, aPDT, and amikacin interact to modulate antimicrobial activity, inflammation, and tissue repair.

## Conclusion

In this pilot in vivo study, aPDT mediated by curcumin-loaded BSA nanoparticles in combination with amikacin showed descriptive findings consistent with a potential therapeutic benefit in MDR P. aeruginosa skin infection. Among the formulations evaluated, NanoBSA − /PDT-ATB was associated with more continuous lesion regression and histological features compatible with ongoing tissue repair, whereas NanoBSA + /PDT-ATB showed more evident descriptive changes in the evaluated cytokine profile. Together with the leukocyte findings, these results suggest that nanoparticles with different surface charges may be associated with distinct inflammatory and tissue responses during treatment. However, because of the small and unequal sample sizes and the absence of direct bacterial load quantification, these findings should be considered exploratory and cannot establish antimicrobial efficacy in vivo. Further studies with adequately powered experimental groups, direct bacterial quantification, and mechanistic evaluation of nanoparticle distribution and host inflammatory responses are required to confirm these observations.

## Supplementary Information

Below is the link to the electronic supplementary material.

Supplementary file1 (DOCX 488 KB)

## Data Availability

No datasets were generated or analysed during the current study.
