# Supplementary material for: Surface charge-dependent effects of BSA-curcumin nanoparticles combined with amikacin in antimicrobial photodynamic therapy: A pilot In Vivo study of multidrug-resistant Pseudomonas aeruginosa skin infection
Source: Lasers Med Sci. 2026 Oct 6;41(1):253. doi: 10.1007/s10103-026-05054-5 (PMC13642459; doi:10.1007/s10103-026-05054-5)
Supplement: Supplementary file 1 [file 10103_2026_5054_MOESM1_ESM.docx]

**Surface Charge-Dependent Effects of BSA-Curcumin Nanoparticles Combined with Amikacin in Antimicrobial Photodynamic Therapy: A Pilot In Vivo Study of Multidrug-Resistant Pseudomonas aeruginosa Skin Infection**

**Supplementary Material**

**Table of Contents**

- **Figure S1…………………………………………………………..………………………………………………………..1**
- **Table S1………………………………………………………………………………………………………………………1**

**Figure S1.** UV-Vis spectrophotometric analysis of curcumin. (A) Absorption spectra of curcumin solutions in ethanol at concentrations ranging from 1 to 5 µmol/L. (B) Representative calibration curve obtained from absorbance values measured at 430 nm as a function of curcumin concentration.

Com base nos dados de calibração, a curcumina foi quantificada nas formulações tópicas. A formulação de curcumina livre apresentou concentração de 0,71 mmol/L, correspondente a 6,9% (m/m). As formulações NanoBSA− e NanoBSA+ apresentaram concentrações de curcumina de 3,8 e 2,7 µmol/L, correspondendo a 0,0033% e 0,0029% (m/m), respectivamente (Tabela S1). Assim, as duas formulações NanoBSA continham curcumina dentro da mesma faixa de concentração

**Table S1**. Quantification of curcumin in the topical formulations.

|  | Free Cur | NanoBSA- | NanoBSA+ |
| --- | --- | --- | --- |
| Curcumin concentration | 0,71 mmol L^-1^ | 3,8 µmol L^-1^ | 2,7 µmol L^-1^ |
| Content m/m (%) | 6,9 | 0,0033 | 0,0029 |
